# Anthropogenic Impacts on Coral-Algal Interactions of the Subtropical Lagoonal Reef, Norfolk Island

**DOI:** 10.1093/iob/obaf004

**Published:** 2025-02-10

**Authors:** M L Ho, C Page, B Leggat, T Gaston, S Eckhardt, T Ainsworth

**Affiliations:** Centre of Marine Science and Innovation, School of Biological, Earth & Environmental Sciences, University of New South Wales, Sydney, Kensington, New South Wales 2052, Australia; Centre of Marine Science and Innovation, School of Biological, Earth & Environmental Sciences, University of New South Wales, Sydney, Kensington, New South Wales 2052, Australia; School of Environmental and Life Sciences, The University of Newcastle, 10 Chittaway Road, Ourimbah, NSW 2258, Australia; School of Environmental and Life Sciences, The University of Newcastle, 10 Chittaway Road, Ourimbah, NSW 2258, Australia; School of Environmental and Life Sciences, The University of Newcastle, 10 Chittaway Road, Ourimbah, NSW 2258, Australia; Centre of Marine Science and Innovation, School of Biological, Earth & Environmental Sciences, University of New South Wales, Sydney, Kensington, New South Wales 2052, Australia; Centre of Marine Science and Innovation, School of Biological, Earth & Environmental Sciences, University of New South Wales, Sydney, Kensington, New South Wales 2052, Australia

## Abstract

Reef building corals are important in subtropical marine ecoregions, shaping ecosystems and providing habitats for fish and benthic species. Algal communities contribute substantially to the benthic population structure across subtropical coral reefs, however increasing algal cover on subtropical reefs is also linked to degraded ecosystems as has been shown on tropical systems. As such, the dynamics of coral-algal interactions on subtropical reefs are also likely to be an indicator of ecosystem health on subtropical ecosystems. The subtropical lagoonal coral reef of Norfolk Island within the Norfolk Marine Park has been impacted by a regime of disturbance since 2020 including flooding, sedimentation, and heat stress events. Assessing the type and extent of algal interactions with the dominant coral *Pocillopora damiconis* within the reef sites of Emily Bay, Slaughter Bay, and Cemetery Bay has the potential to provide insight into drivers of ecosystem decline within the reef. Similarly, photochemical efficiency, as measured by yield (Fv/Fm) using pulse amplitude modulated fluorometry, can be used to provide a measure of the health of corals on reefs during degradation events. Here we assess the extent of coral-algal interactions and health of colonies of *P. damicornis* prior to the onset of summertime conditions (April 2023) and during summertime conditions (December 2023). Seasonal and within site dynamics of coral-algal interactions were evident by a significant bloom of red cyanobacteria (*P* < 0.0001, April 2023) and *Lyngbya* {*P* < 0.01 [Slaughter Bay West (SBW)], *P* < 0.01 [Slaughter Bay East (SBE)], December 2023}. Within reef, variability of coral-algal interactions was most evident for *Lyngbya*, and on the Norfolk reef, interactions of *Lyngbya* with *P. damincornis* were found to be significantly higher at slaughter bay west (SBW 30.2% of interactions) and east (SBE 24.6% of interactions) in December 2023 than at neighboring Emily (11.6% of interactions) and Cemetery Bay (0.6% of interactions). Pulse Amplitude Modulated (PAM) fluorometry also highlighted the potential for algal interactions to influence the photochemical efficiency of *Pocillopora damicornis*. Benthic structure, as measured by coral-algal interactions, and coral health within the Norfolk lagoonal, both highlight the potential for anthropogenic drivers of reef decline to influence the health of the ecosystem. Further investigation is therefore necessary to elucidate the specific causes and consequences of algae linked to poor water quality, such as red cyanobacteria and *Lyngbya*, interacting with corals.

## Introduction

Competition between scleractinian corals and algae is a key process in determining the benthic composition and structure of a coral reef (Karlson 1999; Connell et al. 2004). Understanding the interactions between coral and algae is especially fundamental during phase shifts when reefs shift from coral to algal-dominated communities (Hughes 1994; McCook 2001). Macroalgae such as filamentous turf algae commonly found on coral reefs are associated with benthic dynamics in both healthy and degraded coral reefs and are critical indicators of reef conditions (Adey 1998; Bruno et al. 2014). Some filamentous turf macroalgae provide protection to coral via shading, others could damage corals via abrasions or through overgrowth on lesions (McCook et al. 2001). The increase in turf algae cover coupled with the decline in hard coral cover is considered a main indicator of coral-algal shifts (Hughes et al. 2010).

Naturally, the coverage and diversity of macroalgae varies between space and time due to different factors such as herbivory, competition, and oceanographic processes such as wave action (Steneck and Dethier 1994; Connell et al. 2004). Seasonal changes in temperature and light also contribute to the dynamics of macroalgae on reefs (Glenn et al. 1990; Ateweberhan et al. 2006; Fulton et al. 2014). Nutrient enrichment of the ambient environment coupled with seasonal variation also contributes to changes in the composition of algal communities, as different functional groups respond differently to the changes (Dennison et al. 1999; Albert et al. 2005; Ford et al. 2018; Page et al. 2023b).

Macroalgae can compete with coral for coverage through different mechanisms; these include physical shading and abrasion (Lirman 2001; Box and Mumby 2007), production of chemicals (Rasher et al. 2011), and vectoring pathogens (Nugues et al. 2004b). Coral populations globally have declined by 14% from 2009 to 2018, mirrored by an increase in cover of macroalgae on coral reefs (Souter et al. 2021). However, a recent study found that this correlation may be substantially affected by different benthic species on the reef, such as sea urchins and parrotfish, whilst also indicating that low macroalgal cover may negatively affect coral growth through competitive exclusion (Chan et al. 2023). These studies suggest that comparing coral cover and algal cover separately, and not as a combined analysis, could lead to ambiguous results on coral-algal interactions. Brown et al. (2018) found that a higher frequency of coral-algal interactions is observed to be associated with healthy and heterogeneous coral assemblages on Heron Island. In their study, Brown et al. (2018) also found that the highest occurrence of coral-algal interaction occurred when coral cover was ∼50% and when algal cover was ∼20%, leaving ∼30% of abiotic coverage. Additionally, Brown et al. (2018) found that the increase in algal cover beyond 20% did not contribute to any increment in coral-algal interactions.

Pandolfi et al. (2003) highlighted the risk of reef degradation as a community shifted from coral to algal dominance on reefs in 2003. In the two decades following this study, an increasing body of evidence has highlighted particular algal types as indicators of reef decline including *Lyngbya spp.* (hereafter referred to as *Lyngbya*), red cyanobacterial mats, other turfing algae, and macroalgae (Arnold et al. 2010; Fong and Paul 2011).

Anthropogenic pressures that contribute to reef degradation, such as the removal of herbivorous fishes through overfishing and the reduction of benthic species (Lewis 1986; McManus and Polsenberg 2004; Humphries et al. 2020), destruction of habitat, pollution from terrestrial runoff and sedimental entrapment (Fabricius 2005), can also contribute to the increase in coverage of macroalgae. Given the rapid shift in coral reef ecosystem dynamics measured in the last decade (Hughes et al. 2018), to understand the impact of anthropogenic activities on the competition between coral and macroalgae (Bruno et al. 2014), it is essential to investigate the coral-algal interactions as an indicator of reef dynamics (Connell et al. 2004).

To date, studies focusing on understanding coral-algal interactions mostly demonstrate negative impacts of direct contact, resulting in the reduction of coral growth rate, calcification, fecundity, and survivorship (Tanner 1995; McCook 2001; Jompa and McCook 2003; Rasher and Hay 2010; Ferrari et al. 2012). Certain algae and cyanobacterial mats, such as *Lyngbya majuscule*, have been identified as detrimental to the scleractinian corals. They are known to be harmful on reefs by forming mats of tissue that turn sediment and bottom water anoxic (Albert et al. 2005). While there is a growing body of literature examining coral-algal interactions on tropical reefs (Brown et al. 2018, 2019), few studies have focused on coral-algal interactions in subtropical reef ecosystems. Subtropical reefs are characterized by relatively low coral cover and a higher abundance of fleshy macroalgae (Harriott and Banks 2002). The dominant species and seasonal biomass dynamics of macroalgae are different from tropical coral reefs (Mendes et al. 2015; Graba-Landry et al. 2018). Studies on subtropical coral reefs have found that several algal functional groups are dominant, including crustose coralline red algae, and turfy macroalgae (Vroom and Page 2006; Vroom and Braun 2010). Investigating the effects of subtropical reef coral-algal interactions based on different levels of macroalgae coverage is necessary.

In the current study, coral-algal interactions are investigated at near-shore coral reef sites at high-latitude coral reefs on Norfolk Island, South Pacific. Norfolk Island's inshore reefs are exposed to varying levels of terrestrial runoff inputs and have been experiencing an ongoing coral disease event which is suggested to be related to terrestrial input of dissolved inorganic nitrogen (DIN) from nearby Kingston and Arthur Vale Historic Area (KAVHA) during rain events (Page et al. 2023a). Changes in benthic dynamics of macroalgae have also been observed in recent years (Leggat et al. 2023). Here, we investigate the seasonal dynamics of coral-algal interactions at Norfolk Island and examine the effects of these interactions on the photosynthetic efficiency of the coral host *Pocillopora damicornis*, a common subtropical and tropical reef-building coral (Hoeksema et al. 2014), in April 2023 and December 2023.

## Methods

### Study site

**Fig. 1:**
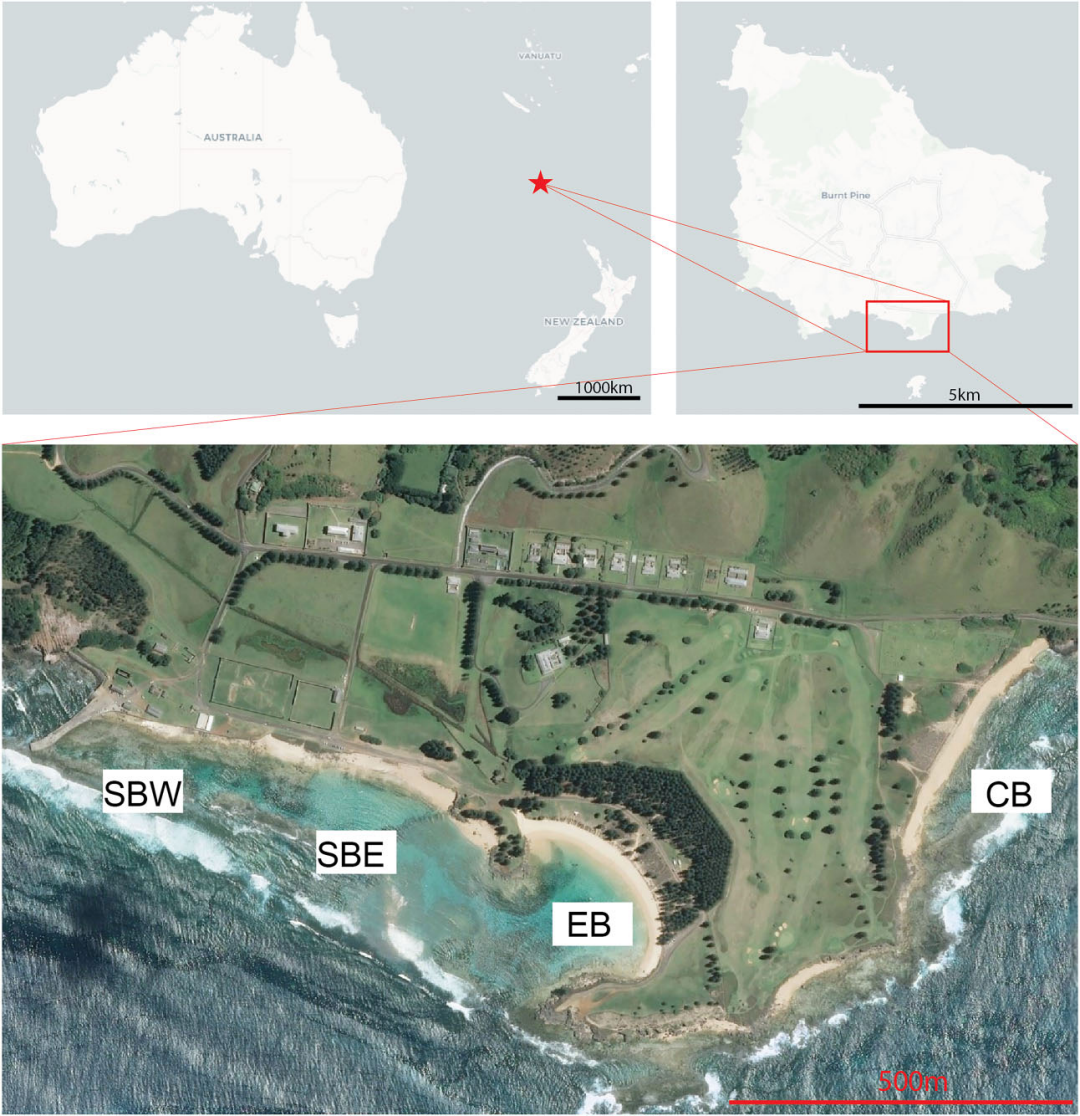
Location of Norfolk Island (top left and right). Location of study sites abbreviations: SBW, Slaughter Bay West; SBE, Slaughter Bay East; EB, Emily Bay; and CB, Cemetery Bay (Bottom). [Positron (Light) and Dark Matter by Carto; World Map by Bing Map].

This study was conducted on the inshore reefs of Norfolk Island, South Pacific (29.06°S, 167.96°E) in April 2023, and December 2023. The island is considered to be marginal as it is situated between both subtropical and tropical marine ecoregions (Spalding et al. 2007; Beger et al. 2014). The reef sites of Slaughter Bay (SB) (divided into West and East as SBW and SBE respectively), Emily Bay (EB), and Cemetery Bay (CB) were studied. SB and EB are lagoonal reefs (Fig. 1) subjected to anthropogenic disturbances such as nutrient run-off from the KAVHA freshwater catchment. Freshwater from this catchment could spill into the lagoon during heavy rainfall. CB is an unconnected lagoonal reef to the east that is presumed to have little impact from land-based pollution due to a lack of observable surface water runoff into this lagoon. SB and EB are divided into three functional sites; as EB features a sewage input directly into the bay, while SBE is exposed to an opening that introduces seawater exchange between the lagoon and outside the lagoon, and SBE is significantly shallower compared to the other three sites. These sites are all located within protected bays on the southern side of Norfolk Island (Fig. 1).

### Rainfall and sea surface temperature data

Sea surface temperature and daily rainfall data are provided for the context of this study from April 1, 2023 to December 31, 2023. The sea surface temperature was obtained from NOAA Coral Reef Watch Australia 5 km Regional Virtual Station (Norfolk Island, Version 3.1). Sea surface temperature provides a satellite-derived night-time measure representation of the surface temperature of the water column for a 5 × 5 km area. Daily rainfall data was obtained from the Bureau of Meteorology Daily Rainfall report of Norfolk Island Aero Station (Station number: 200288, elevation 112 m).

### Assessment of coral-algal interactions

**Fig. 2:**
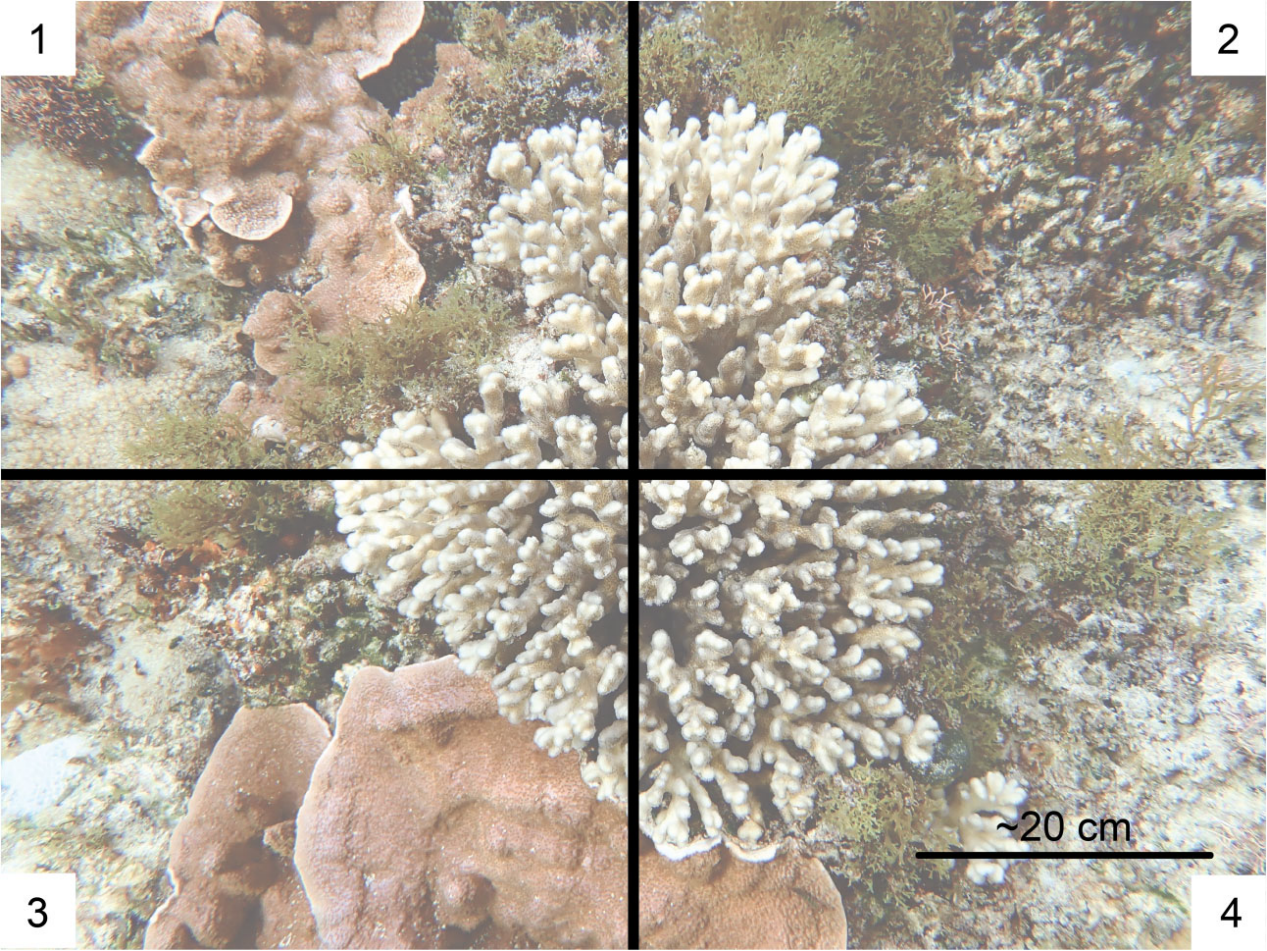
The quadrants of an image. In this example, quadrant 1, 2, and 4 are dominated by *Dictyota*. Quadrant 3 (Bottom left) is dominated by *Montipora* spp; therefore, this colony is interacting with 75% *Dictyota* and 25% scleractinian coral.

Interactions seasonally took place from April 1, 2023 to April 6, 2023, and from December 5, 2023 to December 15, 2023 to observe the seasonal variation of interactions at the beginning of winter and summer, respectively. The surveys were conducted during or just after low tide for accessibility to the reef and safety reasons. Twenty parallel 10 m transect snorkel swims were completed in SBW, SBE, and EB, where starting points were selected haphazardly and not marked for both April and December. For CB where the accessible area is narrower, 38 parallel transects of 5 m were used instead. To avoid double counting, each parallel transect is 4 m apart, therefore, the survey for each site covers approximately 380 m^2^. Each colony of *P. damicornis* was at least 2 m apart to ensure the capturing of individual colonies and within 0.5 m on both sides of the centerline of the transect. Images were photographed with an Olympus TG-6 camera. Photographs of *P. damicornis* were consistently taken from above the coral colonies. The numbers of images of individual colonies taken during the survey periods are shown in Table 1. To analyze the relative occurrence of different types of coral-algal interactions, each photographed colony was overlayed with four quadrants. For each quadrant, the benthic species with the highest area in contact with the coral colony edge is recorded as the dominant algal type (Fig. 2). The results from the analysis are then separated into six major categories of interactions: red cyanobacteria, *Dictyota* spp. (hereafter referred to as *Dictyota*), *Lyngbya* spp. (hereafter referred to as *Lyngbya*), *Caulerpa* spp. (hereafter referred to as *Caulerpa*), scleractinian corals, and other macroalgae (including *Helimeda, Lithophyllum* spp., *Ulva* spp., etc.) for assessment of interactions between algae, coral, or other sessile invertebrates with *P. damicornis*. The total number of quadrants containing each interaction was determined for the site and survey period, and the relative percentage of each interaction group was calculated. The other sessile invertebrate groups were not assessed in this study for statistical significance as they are not known to be competitive with scleractinian corals.

**Table 1:** Number of images of individual colonies taken per site during the survey.

	Slaughter Bay West	Slaughter Bay East	Emily Bay	Cemetery Bay
April	142	46	34	90
December	101	64	56	85

The dominant group interacting with the coral for each photo quadrant was identified, and for each quadrant where they are dominant, 25% was added to the summation of the percentage that group was interacting with the coral (Fig. 2).

### Photosystem II photochemical efficiency

**Fig. 3:**
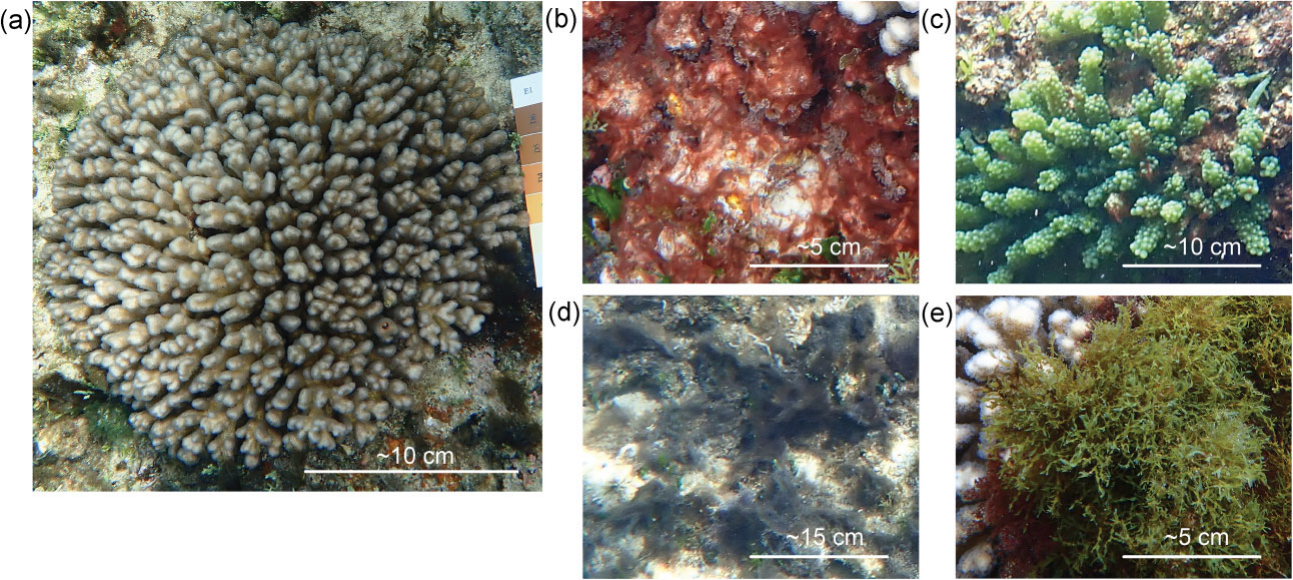
(**a**) The Scleractinian coral, *Pocillopora damicornis*. The four types of benthic algae examined in this study were (**b**) red cyanobacteria, (**c**) *Caulerpa* spp., (**d**) *Lyngbya* spp., and (**e**) *Dictyota* spp.

Four coral-algal interaction groups identified above were established from the dominant algal types identified as interacting with colonies of *P. damicornis* (Fig. 3). Coral fragments were collected from *P. damicornis* colonies interacting with at least 75% of their perimeter in contact with the algae or coral of interest along their perimeter. For each coral-algal interaction group as well as the coral-coral interaction group, four fragment samples from four separate colonies of *P. damicornis* (3–7 cm in length) were collected at three sites (SBW, SBE, and EB) (collection permit PA2021-00012-1) by snorkel and were placed in labelled plastic zip-lock bags held with seawater. Collections took place at low tide and fragments were stored in cool and dark conditions and dark-adapted to allow for the photosynthetic cells to reach a zero non-photochemical quenching state following collection and prior to imaging PAM fluorometry measurement (Murchie and Lawson 2013). Imaging PAM fluorometry measurement was undertaken on land because of the inaccessibility to scuba diving equipment for utilizing monitoring or diving PAM fluorometry measurements. Samples were collected from all sites except for CB due to conservation measures by Australian Marine Parks. The Walz Imaging-PAM M-Series Maxi (Walz Photosynthesis Instrument 2024) was used to record dark-adapted yield from *P. damicornis* samples, and ImagingWin software was used for post-processing of PAM fluorometry data (Walz Photosynthesis Instrument 2024). Photosystem II (PSII) yield was recorded at three different points on the top of each coral branch and averaged. The results were exported, and the average yield (Fv/Fm) for each fragment was calculated and plotted in Rstudio (RStudio Team 2023) using R package ggplot2 (Wickham et al. 2024) for representation in box and whisker plots by interaction group and site.

### Statistical analysis

Data of the percentage of occurrences of macroalgae groups interacting with *P. darmicornis*, were visualized in Rstudio using R package ggplot2 (Wickham et al. 2024) and statistically tested using R package emmeans (Lenth 2024).

Pairwise differences in relative abundance of interaction groups among locations and photochemical efficiency values were tested in this section for statistical analysis.

Chi-squared test and Fisher's exact test were used to determine the difference between two time points in the relative abundance of coral-algal interaction groups and to determine differences between locations within the same time point. The percentage of dominant interaction groups from the analysis of the dominant algal type was converted into contingency tables with proportions of abundance against time points. The expected frequencies for each contingency table were calculated using the Chi-squared test. In the case where the expected frequency is below 5, Fisher's exact test was used. *P*-values were adjusted for multiple testing using Bonferroni correction (alpha value = 0.05, threshold value = 0.01) to account for the increased risk of Type I error when making multiple statistical tests (Dunn 1961; Armstrong 2014). Principal component analysis is used to show the likeliness of the presence of each algal or coral group interacting with *Pocillopora damicornis* for the images taken.

Prior to analysis, data exploration and model validation were performed using plots of residuals vs. fitted values. One-way ANOVA was performed for post-hoc tests to identify pairwise differences in the relative abundance of interaction groups among locations to accommodate for the absence of some data from the photochemical efficiency measurement. The photochemical efficiency values obtained from the two time points (April and December) were analyzed separately and compared. Photochemical efficiency values were fitted to linear models for post-hoc tests, and the data were not transformed for these tests. No major assumptions were violated in this ANOVA and post-hoc test.

## Results

### Rainfall and sea surface temperature data

**Fig. 4:**
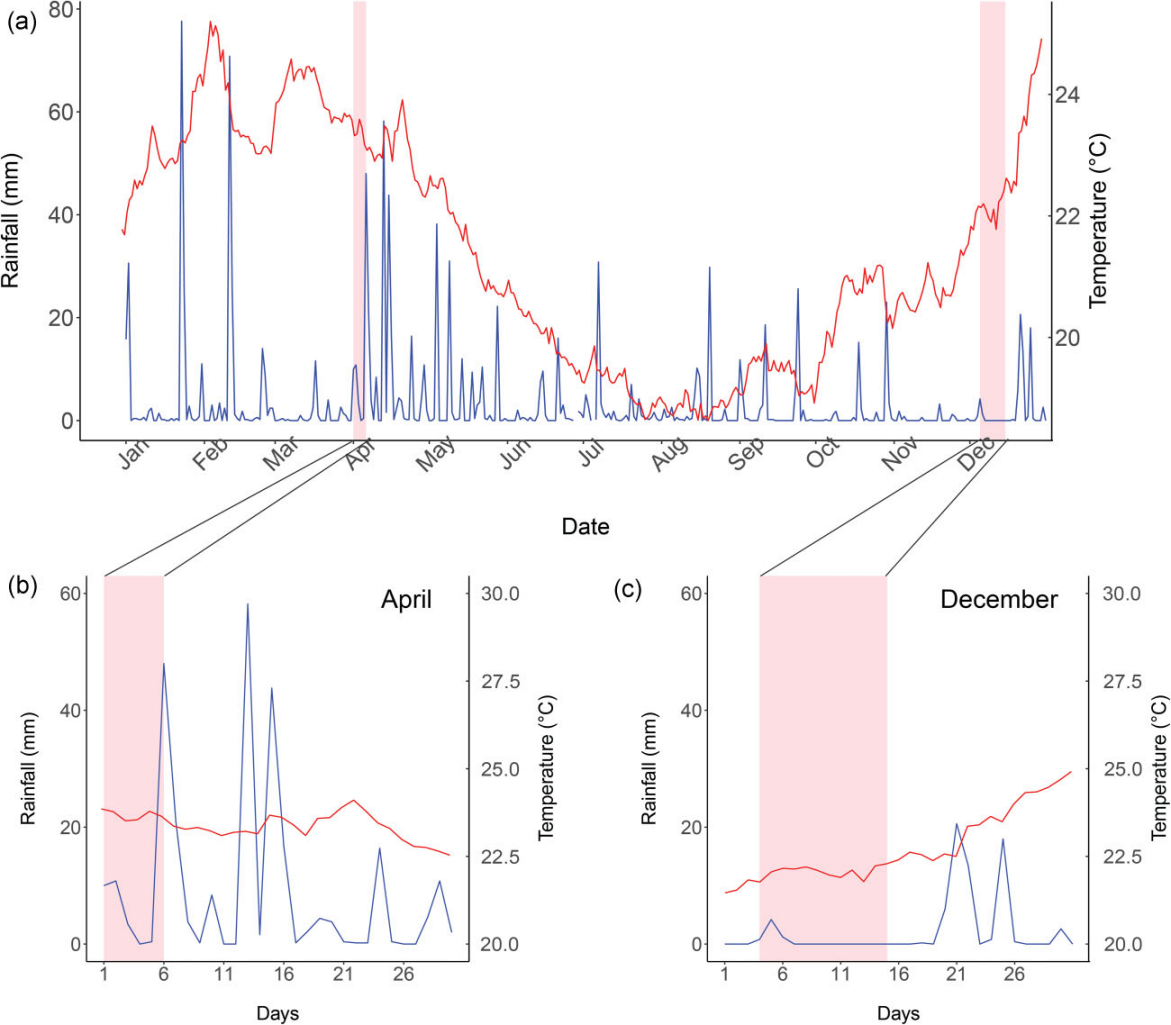
(**a**) Sea surface temperature (red line) and annual rainfall (blue line) of Norfolk Island in 2023. (**b**) Monthly recorded sea surface temperature (red line) and monthly rainfall (Blue line) of April 2023. (**c**) Monthly recorded sea surface temperature (red line) and monthly rainfall (Blue line) of December 2023. Pink boxes highlighted the period of survey.

No flooding was reported prior to the first in-situ survey (1st April–6th April), the second survey (4th December–15th December), and any time between the two survey periods (Leggat et al. 2023). During April 2023 (Fig. 4b), there was a recorded monthly total of 272 mm total rainfall with a peak rainfall of 58.2 mm, recording the highest level of rainfall by month throughout the year. Relatively low rainfall was recorded in December (Fig. 4c), the maximum daily rainfall recorded was 20.6 mm with a monthly total of 68.2 mm. Sea surface temperatures were recorded throughout the year, with a monthly average of 23.18°C in April and 22.75°C in December 2023.

### Assessment of coral-algal interactions

**Fig. 5:**
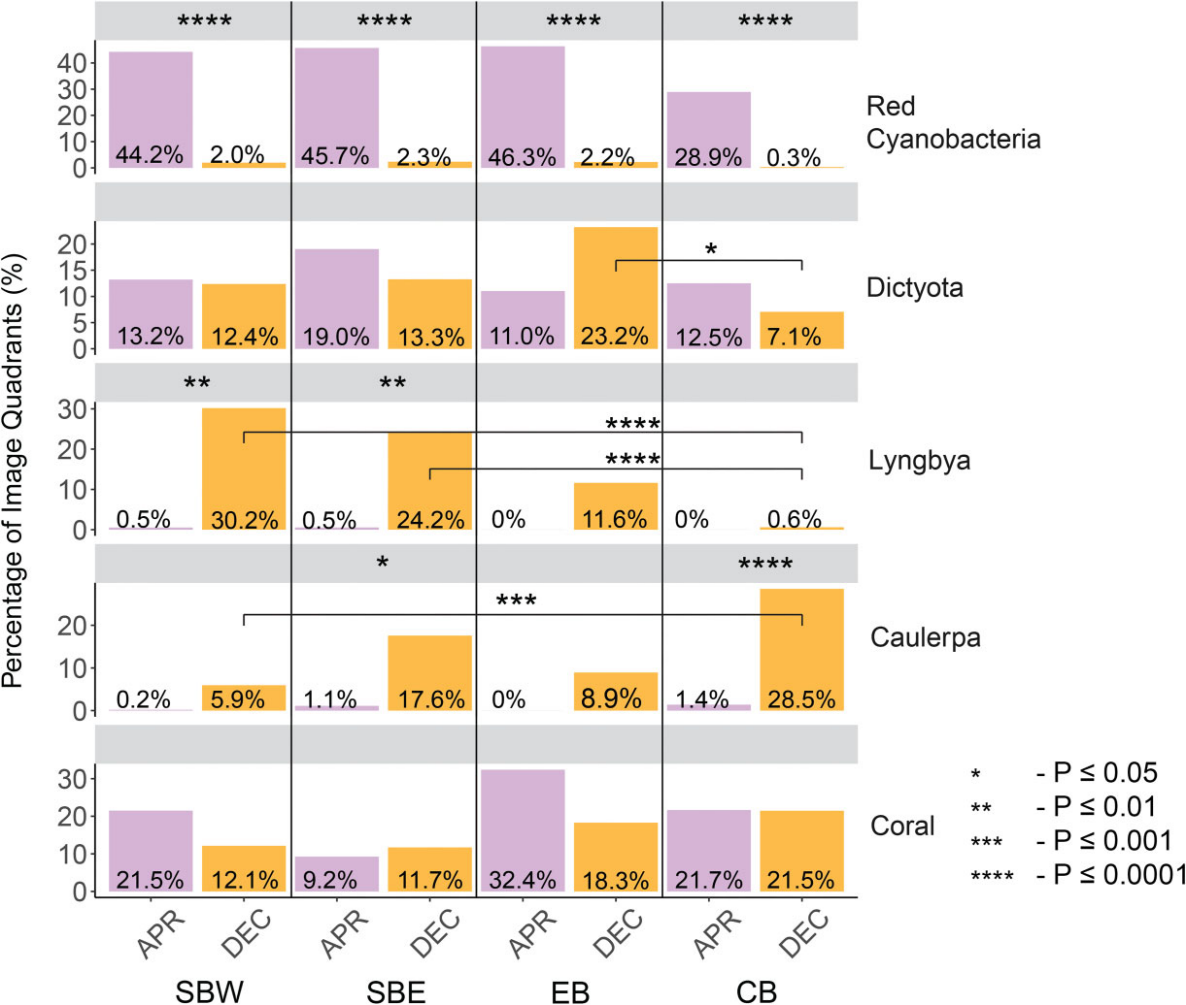
The percentage of image quadrants occupied by each type of coral-algal or coral-coral interaction. Bars on the left of each plot denote April's coverage, bars on the right of each plot denote December's coverage. Asterisks in horizontal bars represent the differences between coverage between time point by location. *P*-values are adjusted using Bonferroni correction.

The percentage of occurrence for each coral-algal interaction group (Fig. 5) differed throughout the sites and seasons. In general, the red cyanobacteria group is found to have a high percentage in April 2023, with a significant difference between the two survey time points in SBW (χ^2 ^= 51.66, *P* < 0.0001), SBE (χ^2 ^= 28.25, *P* < 0.0001), EB (χ^2 ^= 24.04, *P* < 0.0001), and CB (χ^2 ^= 25.84, *P* < 0.0001). The percentage occurrence of the *Dictyota* group observed between April and December were not found to have any significant effect based on time points of surveys. The percentage of occurrence of *Lyngbya* is seen to be higher in December than in April with differences in SBW (χ^2 ^= 12.18, *P* < 0.01) and SBE (χ^2 ^= 10.37, *P* < 0.01) only. The presence of the *Caulerpa* group in December is significantly higher than in April in CB (χ^2 ^= 23.73, *P* < 0.0001).

In April, there were no significant differences in each coral-algal interaction group by location. Variation across sites was observed in December for the *Dictyota* coral-algal interaction group between EB and CB (χ^2^ = 7.46, *P* < 0.1). Significant differences were also observed for the *Lyngbya* coral-algal interaction between SBW and CB (χ^2 ^= 27.04, *P* < 0.0001), as well as between SBE and CB (χ^2 ^= 18.88, *P* < 0.0001). A difference in the percentage occurrence of the *Caulerpa* group was observed between SBW and CB (χ^2 ^= 15.68, *P* < 0.001) with the presence being higher in CB.

Principal component analysis (PCA) is used to measure the relationship among the presence of algal groups across all images taken at the sites. For April (components = 5), the total variances of the relationships between the presence of different algal groups across all four sites are explained by the first two principal components (SBW: 60.5 and 30.9%; SBE: 59.1 and 23.7%; EB: 73.2 and 23.5%; CB: 53.1 and 30.9%). Similarly, the total variances for December (components = 5) across all sites are explained by the first two principal components (SBW: 49.9 and 24.8%; SBE: 48 and 27.9%; EB: 39.3 and 31.9%; CB: 63.8 and 26.3%) (Fig. S1).

**Fig. 6:**
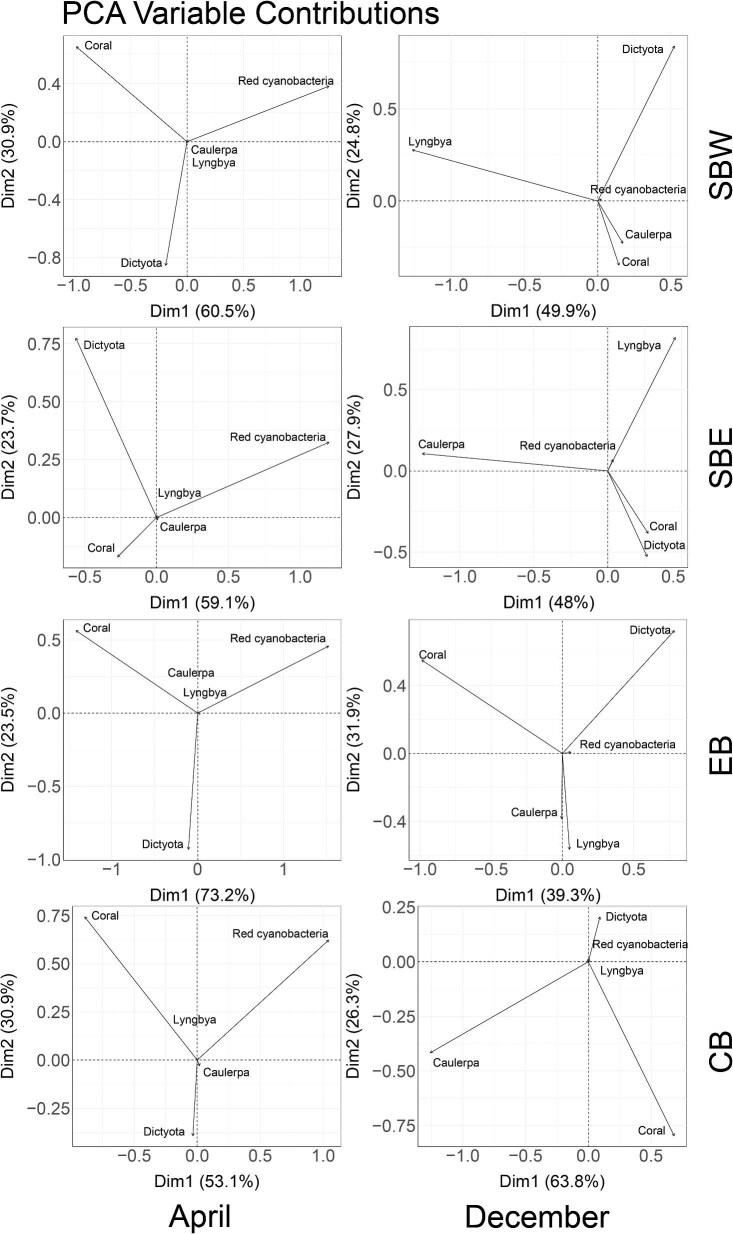
The principal component analysis (PCA) variable contributions of presence of algal groups from images across all sites in April and December.

The PCA variable contributions of the presence of algal groups for images taken across all sites in April and December revealed the contribution of each observed group towards the principal components (Fig. 6). In April, the interaction groups of coral, *Dictyota*, and red cyanobacteria were observed to be the main contributors to the principal components; while the remaining two groups, *Caulerpa* and *Lyngbya* had little to no contribution to the principal components. For December, *Dictyota, Lyngbya*, coral, and *Caulerpa* are seen as the main contributors to the principal components in all sites except for CB, where *Lyngbya* had little to no contribution to the principal components. In contrast to April, red cyanobacteria had little to no contribution to the principal components across all sites.

### Photosystem II photochemical efficiency

**Fig. 7:**
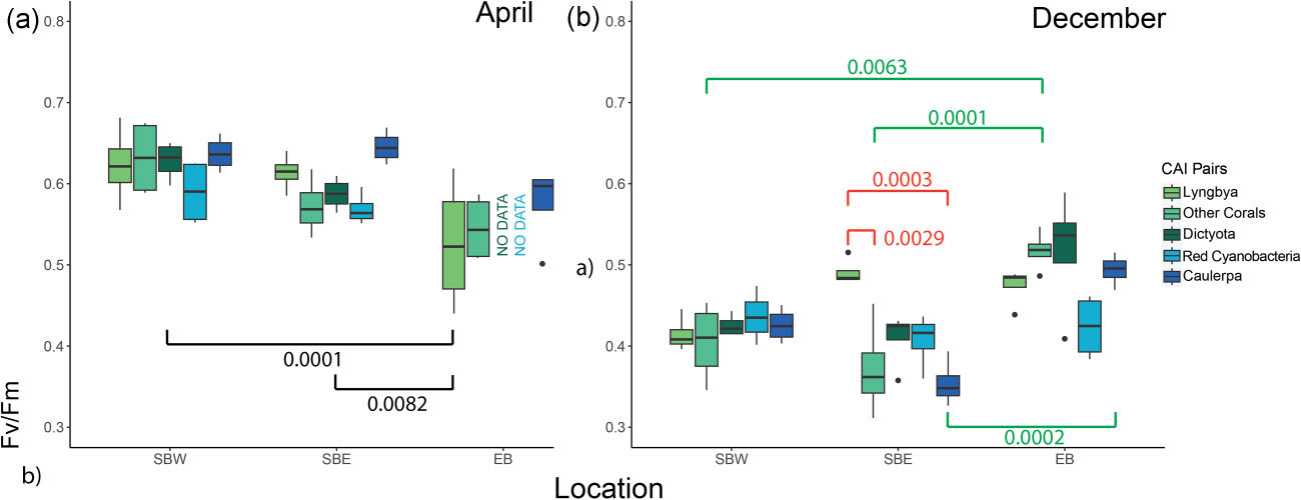
(**a**) PAM fluorometry results from April 2023. The data for Dictyota and red cyanobacteria were corrupted and are missing from April's photosystem II yield plot. Black values indicate significant difference between sites. (**b**) PAM fluorometry results from December 2023. Green values indicate the significant difference between sites within the same group. Orange values indicate the significant difference between coral-algal interactions groups within the same location.

Results of photochemical efficiency analysis and the environmental parameters can be found in Table S1. The values collected from April indicated significant differences in photochemical efficiencies between sites only, but not between coral-algal interaction groups and within sites (on numerator of 2 and denominator DF of 49, *F*-statistic = 11.03, *P* < 0.001). One-way ANOVA of the linear model showed a significant difference in PSII yields in April between the three study sites of SBW, SBE, and EB (Fig. 7a). Post-hoc tests revealed the difference between SBW and EB (*P* = 0.0001), and SBE and EB (*P* < 0.01).

There were strong relationships between coral-algal interaction groups and locations, within interaction groups, and within locations in December. The data were fitted to a linear model (on numerator of 14 and denominator DF of 45, *F*-statistic = 7.449, *P* < 0.0001). Significant differences were revealed using one-way ANOVA on the linear model between study sites in December (Fig. 7b). A post-hoc estimated marginal means test revealed the difference in mean photochemical efficiency are significant between SBW and EB (*P* < 0.0001), and between SBE and EB (*P* < 0.0001). Significant differences for the same coral-algal interaction groups between different locations are identified in the post-hoc estimated marginal means test; in the coral-coral group, the difference of photosystem ii yield values is significant between SBW and EB (*P* < 0.01), and between SBE and EB (*P* = 0.0001). Significant difference was also observed for one of the coral-algal interaction groups, *Caulerpa* between SBE and EB (*P* < 0.001). There were no significant differences between other coral-algal interaction groups between locations. In SBE, significant differences between PSII values were found between the coral-algal interaction groups of *Lyngbya* and coral (*P* < 0.01), a significant difference in mean photochemical efficiency was also recorded between *Lyngbya* and *Caulerpa* (*P* < 0.001); in both cases *Lyngbya* was observed to have higher mean photochemical efficiency. No other significant differences were observed within a location or for each coral-algal interaction group between locations.

## Discussion

Coral-algal interactions are important in the benthic dynamics of subtropical coral reefs (Vroom and Braun 2010; Brown et al. 2018). In the current study, the relative occurrence of four coral-algal interactions with *Pocillopora damicornis* and coral-coral interactions with *Pocillopora damicornis* in the subtropical Norfolk Island inshore reefs were examined in April 2023 and December 2023, and the impact of direct contact between coral-algal interaction groups of *P. damicornis* on PSII photochemical efficiency (F_v_/F_m_) was measured over two time points. The higher occurrence of coral interactions with red cyanobacteria in April, and *Lyngbya* in December suggests that the composition of cyanobacterial turfs at Norfolk Island varies seasonally. While other interactions also show variation in composition seasonally, the seasonal differences for red cyanobacteria and *Lyngbya* interacting with *P. damicornis* are significantly greater than other interaction groups. Sites differed in the relative occurrences of coral-algal and coral-coral interactions observed on the reefs, as well as short-distance coral-algal interactions, with the occurrences of *Lyngbya* interacting with *P. damicornis* being significantly lower in CB when compared to SBW and SBE in December 2023. PSII yields on collected *P. damicornis* samples in direct contact with algae or coral varied across sites, with the *Dictyota* group in EB displaying the highest mean photochemical efficiency compared to other groups and sites.

### Seasonal variation in coverage and relative occurrence of interaction groups

The analysis revealed a significant difference between April and December on the dominance of red cyanobacteria interacting with *P. damicornis* across all locations. Fourty-six percent of colony quadrants in April were dominated by red cyanobacteria, while in December only ∼2.5% of colony quadrants were dominated by red cyanobacteria. Observations of red cyanobacteria bloom had been recorded on Norfolk Island at the end of Austral summer 2021 (Leggat et al. 2023). Several studies have linked red cyanobacterial blooms to land-based pollution (Paul 2008; Ford et al. 2018; Page et al. 2023a). The occurrence of red cyanobacteria was lower in the bay and it was less likely to be exposed to runoff.

*Lyngbya* interacting with *P. damicornis* in December in SBW and SBE are significantly higher than in April, with 30.2% of colony quadrants in December dominated by the presence of *Lyngbya*. A lower abundance of *Lyngbya* interacting with *P. damicornis* in December was recorded in EB and CB, with just 11.6 and 0.6% of colony quadrants interacting with *Lyngbya*. Blooms of *Lyngbya* in marine ecosystems have also been linked to inshore pollution and heat stress (Watkinson et al. 2005). *Lyngbya spp.* requires a higher concentration of iron in water columns for blooming (Albert et al. 2005; Ahern et al. 2006), which is observed in more anthropogenically-influenced bodies of water. This agrees with our observations and assumptions of significantly lower relative occurrence of *Lyngbya* in CB when compared to the other sites. Additionally, different levels of rainfall recorded in the two research durations may explain the differences in abundance of *Lyngbya* between seasons.

The percentage occurrence of *P. damicornis* interacting with *Dictyota* and other scleractinian corals remained largely consistent throughout the two time points across the reef sites studied. The interactions between *Caulerpa* and *P. damicornis* vary seasonally by site and can be observed in higher relative occurrence in December 2023 at CB. A higher abundance of *Caulerpa* was observed in CB when compared to all other sites. Previous studies and reports observed that the DIN in SB and EB are in general higher than CB due to terrestrial runoffs (Leggat et al. 2023; Page et al. 2023a). In a study on *Caulerpa lentillifera*, the uptake of nitrogen as nutrients resulted in a higher growth rate of *Caulerpa lentillifera* (Liu et al. 2016). An investigation of DIN uptake by *Caulerpa* across the reefs may explain the difference between the percentage occurrences of *Caulerpa* in the inshore reefs of Norfolk Island.

The principal component analysis on understanding relationships between the presence of algal groups for the two time points through the images taken revealed an interesting pattern. In April, the abundance of red cyanobacteria from our images is strongly correlated with the first principal component across all sites, while the abundance of coral shows correlations with the second principal component in three sites (SBW, EB, and CB). *Dictyota* showed strong correlation with the second principal component in SBE. The contribution of *Caulerpa* and *Lyngbya* to the principal components was negligible across all four sites in April. In December, Lyngbya showed high correlation to the first principal component in SBE. *Dictyota* showed strong correlation to the first principal component in SBW, SBE, and EB, it also showed strong correlation to the second principal component in SBW and EB, while red cyanobacteria contributed little (SBE and EB) and had negligible contribution to the principal components (SBW and CB). Studies suggested that the presence of *Lyngbya* is more dominant during summer (Dennison et al. 1999; Albert et al. 2005), and the presence of *Dictyota* is relatively consistent for April and December (Leggat et al. 2023). There was evidence that elevated nutrients combined with herbivory also negatively affected the growth of *Dictyota* spp. (Ramseyer et al. 2021), providing some explanation for the negative relationship in the presence of *Dictyota* and *Lyngbya* in December. More research shall be done on Norfolk Island to confirm the cause of this change in relationship between the presence of *Dictyota* and *Lyngbya* in April and December.

A limitation of this analysis was the potential misidentification of the “dominant” interaction group in a quadrant due to the complexity of benthic composition, or in cases where two or more benthic species are very similar in the proportion of contact to the coral. While out of scope for this study, implementation of artificial intelligence segmentation tools (Kirillov et al. 2023) could be applied to quantify the actual composition of each image.

### Photosystem II photochemical efficiency variations

Previous studies have identified coral diseases in the inshore reefs of Norfolk Island (Page et al. 2023b), further examination of coral structures in *P. damicornis* is required to determine the health of the *P. damicornis* in the inshore reefs of Norfolk Island. These factors may have contributed to the photochemical efficiency variations recorded in this study. Therefore, the results may be further explained if these data were made available and multivariate analysis of the interactions were explored. There are potentially some variations within sites, where these variations could only be reflected through fine-scale measurements of the environmental parameters. This could potentially improve the understanding of how these fine-scale environmental parameters are affecting the health of the corals subjected to coral-algal interactions.

The Fv/Fm values obtained in this study may only reveal a portion of how direct coral-algal interaction could affect *P. damicornis*. Further analysis through methods such as rapid light curve measurements could provide further assessment of coral health under direct coral-algal interaction in *P. damicornis* (Ralph et al. 1999; Ralph and Gademann 2005). Rapid light curve measurements have been used extensively on aquatic organisms for their capability of understanding states of photoacclimation and responses to different light conditions and have been shown to be able to provide more in-depth results on photochemical efficiency and other parameters such as chlorophyll fluorescence (Jesperson and Xiao 2022).

Results from corals collected in April showed varying photosystem ii yield values between interaction groups. Measurements of Fv/Fm values from different sample groups within each site were hypothesized to be similar; however, the results from our studies have shown that some interaction groups, such as *Lyngbya* in SBE during December, and *Caulerpa* in SBE during April, have significantly higher values compared to other groups. Additionally, site differences were also observed for some interaction groups between sites. In December, the Fv/Fm values recorded from Lyngbya in both SBE and EB are the highest compared to other groups. This is surprising as previous studies on *Porites lutea* found that *Lyngbya* could reduce the growth and the Fv/Fm values of *P. lutea* via abrasion when the two are in direct contact (Titlyanov et al. 2007).

What is more interesting is that in December, the interaction group of *Caulerpa* in two sites (SBE and EB) showed lower Fv/Fm values. Present literature studying coral-algal interactions between *Caulerpa* and scleractinian corals identified that the density content of dinoflagellate endosymbionts decreased when the two benthic entities are in direct contact, and this effect is observed to a higher extent when the ambient temperature is elevated (Fu et al. 2023).

From the December PSII yield data, SBW showed consistently low values throughout the four coral-algal interaction groups and the coral-coral interaction group. It adheres to the assumption that SBW would be least influenced by anthropogenic pollution within the lagoon compared to SBE and EB (Leggat et al. 2023; Page et al. 2023a). Worsening water quality caused by rainfall and associated runoff may be associated with coral degradation and disease outbreaks (Haapkylä et al. 2011), while anthropogenic influences such as sewage input may also contribute to the higher photochemical efficiency measurements in April compared to December due to higher rainfall in April. PSII yields measured in EB were in general higher than the measurements from samples obtained from SB, despite that EB is presumed to be the most impacted by terrestrial pollution and disease events (Leggat et al. 2023; Page et al. 2023a). Enrichment of nutrients such as ammonium, compounds of ammonium and phosphate, has been reported to negatively impact the photosynthetic efficiency of corals (Dubinsky et al. 1990). However, a review by Nalley et al. (2023) identified no negative impact to photosynthetic efficiency of corals as a result of exposure to DIN and dissolved inorganic phosphate (DIP), however, elevated levels of DIP has a negative linear effect on photosynthetic efficiency of corals. Additionally, the introduction of nutrient enrichment might positively impact the physiological performance of zooxanthellae, provided that essential nutrients are readily available (Wiedenmann et al. 2013). These results from our research presented the effects of algal-coral interactions and coral-coral interactions on *P. damicornis* on four different sites, and how some of the recorded algal-coral interactions may be detrimental to *P. damicornis*.

Due to the lack of laboratory equipment available on the research site, the collected coral fragments were kept in a non-laboratory environment prior to PAM fluorometry measurement. The authors attempted to keep the samples in a large volume of seawater within an enclosed container to keep the pre-measurement condition constant for all fragments. This method might have reduced the dissolved oxygen within the container and led to decreases in measurement of photochemical efficiency. Use of an in-situ measuring device on controlled coral colonies could provide a baseline for the measurement of photochemical efficiency for more effective comparison of measured values. Future work using in-situ measurements of PSII photochemical efficiencies could eliminate the limitations of lack of lab equipment. Additionally, because of the limited environmental data available, the effect of temperature, rainfall, oceanographic properties, and anthropogenic-induced nutrient enrichment were not studied. Further incorporation of environmental data could provide further insight into understanding the influences of coral-algal and coral-coral interactions.

## Conclusions and future directions

Turfing communities interacting with *P. damicornis* generally have a strong change in abundance between seasons, with the blooming of red cyanobacteria in April 2023, a decrease in cover of red cyanobacteria and blooming of *Lyngbya* in December 2023. Furthermore, the results on Fv/Fm of the *P. damicornis* colonies interacting with the macroalgae groups showed that some coral-algal interactions are detrimental to *P. damicornis* by decreasing the Fv/Fm values of coral branches, and there are possibly more factors, such as environmental parameters and anthropogenic intervention dominating how coral-algal interaction could impact coral health. While further research is needed to elucidate the impacts of interactions on coral function, our results indicate variation in influence on photosystem yield between sites and interaction groups. Future studies could look to conduct PAM fluorometry on the same colonies over different time points to provide comparisons between seasons. Our results indicate that understanding coral-algal interactions and potential phase shifts on subtropical reefs is complicated and is likely influenced by multiple external factors. Further long-term monitoring of the reef condition will be necessary for future studies to assess the potential occurrence of phase shifts and effects of coral-algal interactions on *P. damicornis* on subtropical coral reefs like Norfolk Island.

## Supplementary Material

obaf004_Supplemental_File

## Data Availability

Data and codes for this research is available on https://github.com/MLH95/Ho-et-al-2024_Coral-Algal-Interactions
