# Supplementary material for: Anthropogenic Impacts on Coral-Algal Interactions of the Subtropical Lagoonal Reef, Norfolk Island
Source: Integr Org Biol. 2025 Feb 10;7(1):obaf004. doi: 10.1093/iob/obaf004 (PMC11851010; doi:10.1093/iob/obaf004)
Supplement: obaf004_Supplemental_File [file obaf004_supplemental_file.docx]

**Supplementary File 1**

This supplementary file contains the Supplementary Table 1 and Supplementary Figure 1 for the publication of **Anthropogenic Impacts on Coral-Algal Interactions of the Subtropical Lagoonal Reef, Norfolk Island**, submitted to Integrative Organismal Biology.

This table describes the Fv/Fm results measured from the Pocillopora damicornis samples collected in April and December 2023. The rainfall data and temperature data as context are also provided in this table.

The figure describes the scree plot on the principal component analysis of the images taken over the four sites on the five interaction groups.

Supplementary Table 1. Results of photochemical efficiency recorded and environmental parameters during the study periods. Environmental parameters here provide the background information of the site during the two survey periods.

| Emily Bay | Interaction Group | Fv/Fm | Rainfall | Sea Surface Temperature |
| --- | --- | --- | --- | --- |
| April 2023 | Dictyota | N/A | Mean: 12.1mm  Min: 0mm  Max: 48mm | Mean: 23.49°C  Min: 22.81°C  Max: 23.82°C |
|  | Caulerpa | Min: 0.498  Max: 0.628  Average: 0.575 |  |  |
|  | Lyngbya | Min: 0.413  Max: 0.623  Average: 0.526 |  |  |
|  | Red Cyanobacteria | N/A |  |  |
|  | Coral | Min: 0.466  Max: 0.612  Average: 0.545 |  |  |
| December 2023 | Dictyota | Min: 0.38  Max: 0.593  Average: 0.518 | Mean: 0.41mm  Min: 0mm  Max: 4.2mm | Mean: 22.04°C  Min: 21.44°C  Max: 22.46°C |
|  | Caulerpa | Min: 0.433  Max: 0.538  Average: 0.494 |  |  |
|  | Lyngbya | Min: 0.425  Max: 0.507  Average: 0.474 |  |  |
|  | Red Cyanobacteria | Min: 0.364  Max: 0.476  Average: 0.424 |  |  |
|  | Coral | Min: 0.449  Max: 0.549  Average: 0.518 |  |  |
| Slaughter Bay East | Interaction Group | Fv/Fm | Rainfall | Sea Surface Temperature |
| April 2023 | Dictyota | Min:0.554  Max: 0.623  Average: 0.587 | Mean: 12.1mm  Min: 0mm  Max: 48mm | Mean: 23.49°C  Min: 22.81°C  Max: 23.82°C |
|  | Caulerpa | Min: 0.605  Max: 0.682  Average: 0.645 |  |  |
|  | Lyngbya | Min: 0.565  Max: 0.652  Average: 0.614 |  |  |
|  | Red Cyanobacteria | Min: 0.537  Max: 0.605  Average: 0.569 |  |  |
|  | Coral | Min: 0.53  Max: 0.638  Average: 0.572 |  |  |
| December 2023 | Dictyota | Min: 0.348  Max: 0.468  Average: 0.410 | Mean: 0.41mm  Min: 0mm  Max: 4.2mm | Mean: 22.04°C  Min: 21.44°C  Max: 22.46°C |
|  | Caulerpa | Min: 0.315  Max: 0.426  Average: 0.354 |  |  |
|  | Lyngbya | Min: 0.368  Max: 0.59  Average: 0.491 |  |  |
|  | Red Cyanobacteria | Min: 0.352  Max: 0.449  Average: 0.407 |  |  |
|  | Coral | Min: 0.294  Max: 0.476  Average: 0.372 |  |  |
| Slaughter Bay West | Interaction Group | Fv/Fm | Rainfall | Sea Surface Temperature |
| April 2023 | Dictyota | Min: 0.588  Max: 0.674  Average: 0.628 | Mean: 12.1mm  Min: 0mm  Max: 48mm | Mean: 23.49°C  Min: 22.81°C  Max: 23.82°C |
|  | Caulerpa | Min: 0.574  Max: 0.675  Average: 0.637 |  |  |
|  | Lyngbya | Min: 0.509  Max: 0.685  Average: 0.623 |  |  |
|  | Red Cyanobacteria | Min: 0.481  Max: 0.664  Average: 0.590 |  |  |
|  | Coral | Min: 0.563  Max: 0.694  Average: 0.632 |  |  |
| December 2023 | Dictyota | Min: 0.4  Max: 0.459  Average: 0.425 | Mean: 0.41mm  Min: 0mm  Max: 4.2mm | Mean: 22.04°C  Min: 21.44°C  Max: 22.46°C |
|  | Caulerpa | Min: 0.398  Max: 0.455  Average: 0.426 |  |  |
|  | Lyngbya | Min: 0.394  Max: 0.461  Average: 0.415 |  |  |
|  | Red Cyanobacteria | Min: 0.394  Max: 0.476  Average: 0.436 |  |  |
|  | Coral | Min: 0.316  Max: 0.48  Average: 0.405 |  |  |


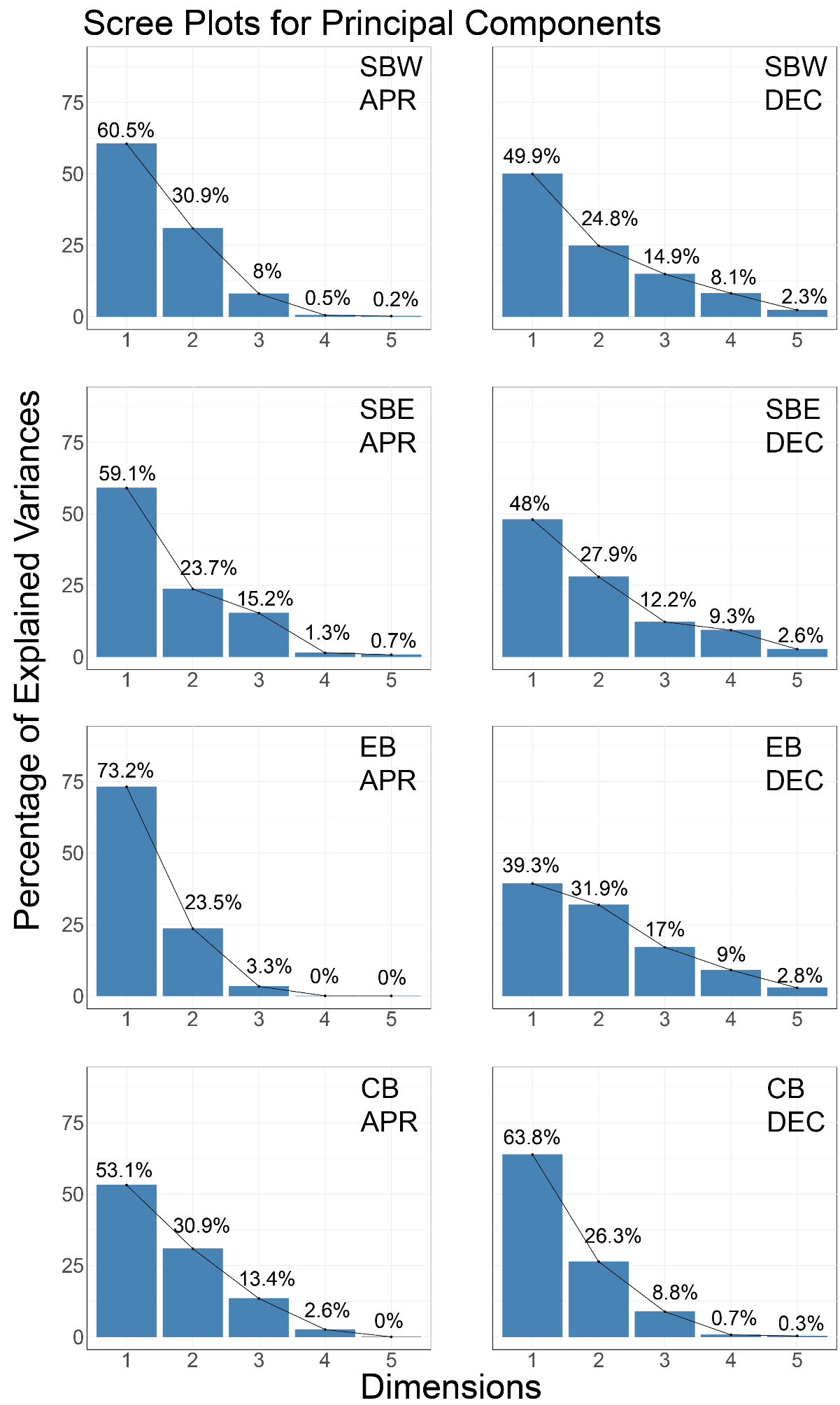


Supplementary Figure 1. Scree plots for principal components for principal component analysis in April and December.
